# Complete median nerve axonotmesis as a late postoperative complication in distal radius fracture

**DOI:** 10.1093/jscr/rjad242

**Published:** 2023-05-12

**Authors:** Sergi Barrera-Ochoa, Jose Antonio Prieto Meré

**Affiliations:** ICATME, Hand and Microsurgery Unit, Hospital Universitari Quiron-Dexeus, Barcelona 08028, Spain; Orthopedic and Traumatology Department, Instituto Guatemalteco de Seguridad Social (IGSS), Guatemala City 01057, Guatemala

**Keywords:** distal radius fracture, median axonotmesis, late complication, volar locking plate, screw migration

## Abstract

Locked volar plate fixation is currently the gold-standard treatment for distal radius fractures. Although volar plating is considered as a reasonably safe treatment option for distal radial fractures, several complications can be observed, such as median nerve injury. We present an 84-year-old male with an intra-articular comminuted fracture of the left distal radius that presented as a late postoperative complication a complete axonotmesis of the median nerve due to screw migration of a locked volar plate. An electromyography was performed confirming complete median nerve axonotmesis, and with proximal stimulation, a Martin–Gruber anastomosis in the proximal forearm was discovered.

## INTRODUCTION

Distal radial fractures are one of the most frequent injuries seen in orthopedic surgery and defined as an indicator of osteoporosis in the elderly.

Locked volar plate fixation has replaced dorsal plating as the treatment of choice for distal radius fractures as it avoids the common complications of dorsal plating, achieving better outcomes [1, 2]. Although volar plating is considered as a reasonably safe treatment option for distal radial fractures, several complications can be observed within this treatment [3]. Median nerve injury after the volar plate fixation of a distal radius fracture is rare.

We present the case of an 84-year-old male, with osteoporosis, who sustained an intra-articular comminuted fracture of the left distal radius and, as a late postoperative complication, suffered a complete axonotmesis of the median nerve due to the screw migration of a locked volar plate.

The patient was informed that data concerning his case would be submitted for publication and agreed to. Patient confidentiality was protected according to the US Health Insurance Portability and Accountability Act.

## CASE REPORT

**Figure 1:**
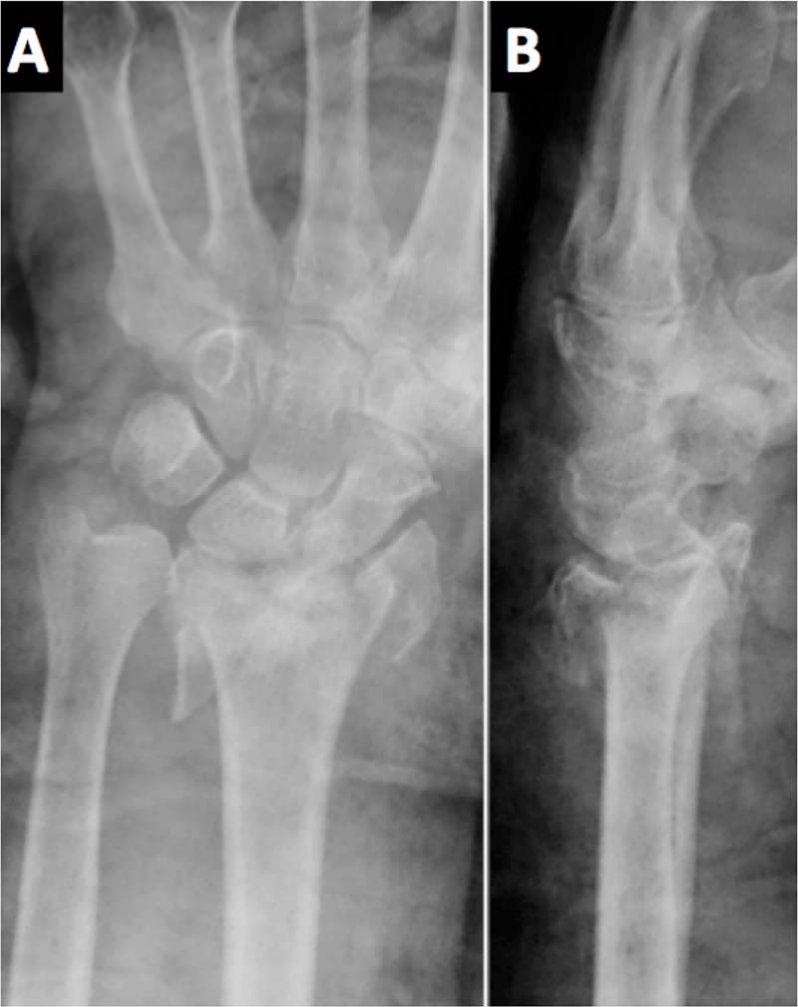
The radiological images in the emergency department showed a comminuted intra-articular displaced fracture of the distal radius.

An 84-year-old, right-handed man attended the emergency room for the pain and swelling of his left wrist after a fall over his outstretched arm from a standing height. An X-ray was performed, showing an intra-articular comminuted fracture on the left distal radius, classified as 23-C3 according to the AO classification (Fig. 1). A cast was placed in the first place; meanwhile, the final treatment was decided.

Several options were discussed for the present patient: first, an open reduction and internal fixation with a volar plate; second, closed reduction and fixation with a K-wire; third, external fixation; fourth, internal fixation with a plate for total arthrodesis; and, fifth, radioscapholunate arthrodesis.

**Figure 2:**
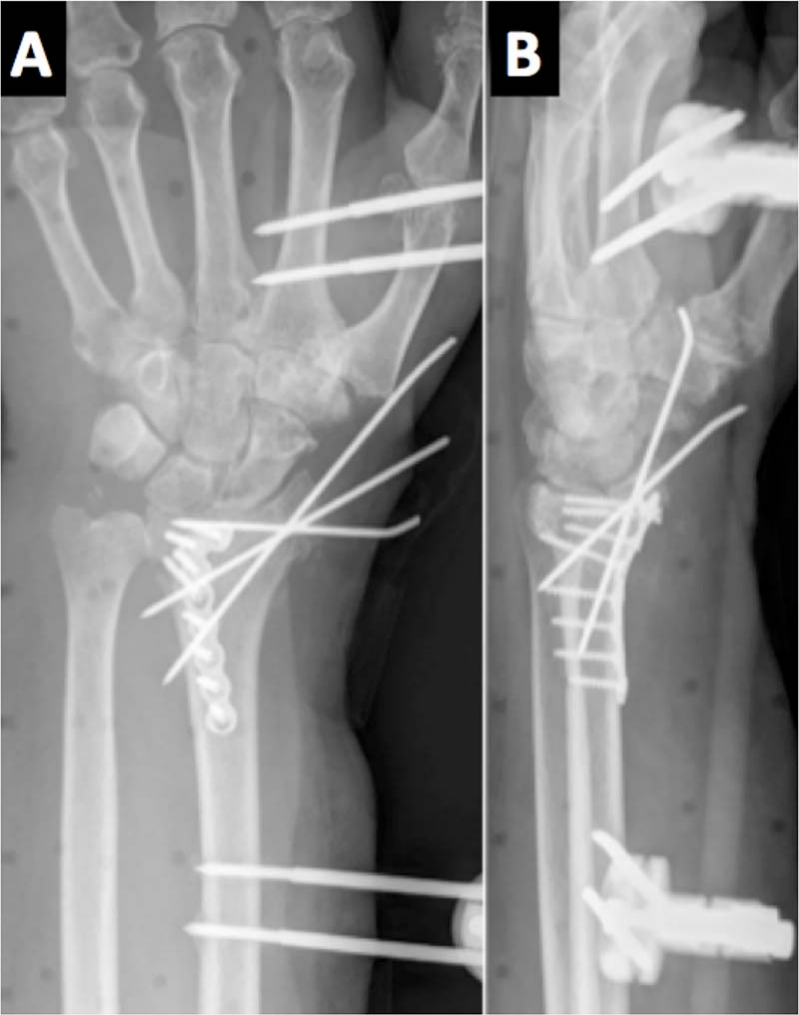
Immediate postoperative radiological assessment.

Due to the comminuted fracture and the bone osteoporosis, the final decision was closed reduction and fixation with K-wires. Due to the inadequate reduction, we decided to associate an external fixator. However, during surgery, an inadequate reduction of the ulnar column was observed, and thus, a mixed synthesis with an L-shaped plate was added (Fig. 2).

**Figure 3:**
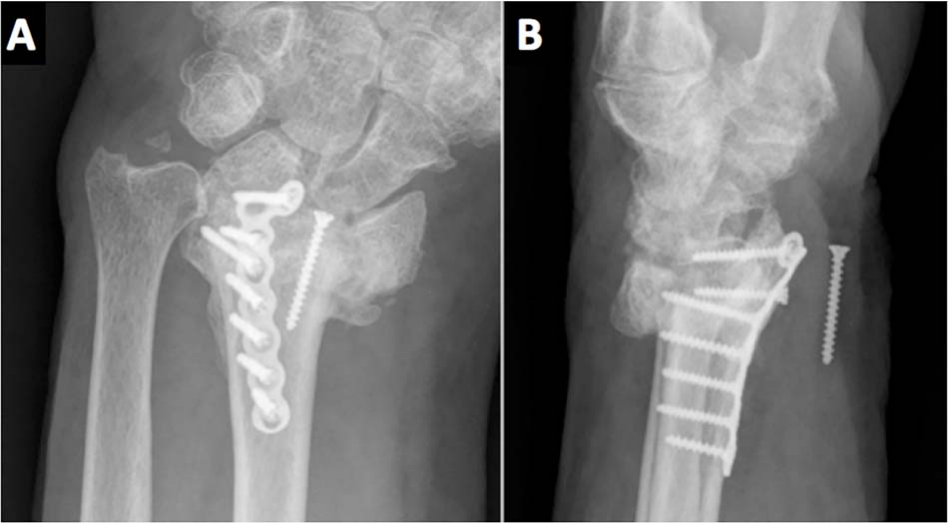
Radiological evidence of a migrated screw lodged in the anterior aspect of the wrist.

On the third month of follow-up, the loosening of the second most distal screw was observed on plain X-rays. Six months after surgery, radiographic controls showed a migrated screw lodged in the anterior aspect of the wrist. Moreover, the patient complained of anesthesia on the median nerve distribution of the hand (Fig. 3). No motor deficits or atrophy of the thenar eminence were noted. A standard electromyography (EMG) was performed resulting in a complete axonotmesis of the median nerve. During the following 8 months, the patient did not attend the follow-ups. Upon his next follow-up visit, the hypoesthesia on the radial three digits persisted despite the fact that no muscular atrophy or motor deficits were observed, as in his last clinical examination. A new EMG was performed again confirming complete median nerve axonotmesis, and with proximal stimulation, a Martin–Gruber anastomosis in the proximal forearm was discovered [4]. The patient refused further surgery for screw removal.

At the 2-year follow-up visit, the fracture had consolidated, both clinically and radiologically. His ranges of movement were 30°–30° of flexion–extension and a radial–ulnar of 10°–15°; all movements were pain-free. His grip strength was 70% of the opposite wrist and he had restarted his job, without complications. The patient continued refusing a new surgery to remove the screw as he was pain-free and performing the activities of daily life without any difficulty.

## DISCUSSION

Locked volar plate fixation for the treatment of distal radius fractures has been the preferred treatment in most fractures since it avoids the complications of dorsal plating [5]. The complications of volar plating in elderly patients have been described previously in the literature [6, 7]. Strictly concerning the median nerve, Nourbakhsh reported median nerve palsy due to fibrosis after a palmar approach for a distal radius fracture [8].

Several studies have described the appearance of carpal tunnel syndrome following a locked volar plate in distal radial fractures, ranging from 1 to 22% [9–11]. Jacubietz et al. described 22% of patients with paresthesia in the median nerve, although all patients recovered not requiring carpal tunnel release [10]. Still, authors defended the palmar plate over the dorsal plate showing the key advantage of the first one: a faster recovery time. In another study group, 1 out of 33 patients included in the study group had to undergo median nerve decompression and plate extraction due to the carpal tunnel syndrome; however, four patients in the external fixator group (*n* = 30) remained with a light sensory deficit, corresponding to a superficial radial nerve branch [11].

Regarding the different surgical procedures available for the current patient; at first, a radio-carpal arthrodesis was considered due to the patient’s osteoarthritis and fracture pattern. Open reduction and internal fixation with a volar plate were also considered. However, despite being the gold-standard treatment for these types of fractures, it was discarded due to the comminuted fracture pattern and the patient’s osteoporosis. Closed reduction and fixation with K-wires was dismissed, as a complete reduction was not achieved, as well as the external fixator. Due to the lack of a congruent joint space and looking for the best functional result possible, an L-shaped plate was associated in the ulnar region. A dorsal bridge plate was also considered, given the good outcomes described in previous studies [12, 13]. However, our group did not have enough experience with this type of fixation, although it should be considered in fractures of the distal radius with metaphyseal and diaphyseal comminution [13]. Distraction bridge plate fixation, especially in these types of fractures, has shown to be safe with minimal complications, with similar functional outcomes to other treatment methods [12].

To our knowledge, a complete axonotmesis of the median nerve as a complication following the volar locking plate has not yet been reported. In our case, the nerve lesion was primarily due to screw migration rather than a complication related with the surgical intervention.

In conclusion, elderly patients with severe osteoporosis or poor bone quality, especially those with a complex fracture line or high-comminuted fractures, the standard treatment should be reevaluated, as uncommon complications could occur.

## References

[1] Lalone EA, Rajgopal V, Roth J, Grewal R, MacDermid JC. A cohort study of one-year functional and radiographic outcomes following intra-articular distal radius fractures. Hand (N Y) 2014;9:237–43. 24839428 10.1007/s11552-013-9586-6 PMC4022950

[2] Ruch DS, Papadonikolakis A. Volar versus dorsal plating in the management of intra-articular distal radius fractures. J Hand Surg Am 2006;31:9–16. 16443097 10.1016/j.jhsa.2005.09.011

[3] Bentohami A, de Burlet K, de Korte N, van den Bekerom MP, Goslings JC, Schep NW. Complications following volar locking plate fixation for distal radial fractures: a systematic review. J Hand Surg Eur 2014;39:745–54. 10.1177/1753193413511936 24262583

[4] Uchida Y, Sugioka Y. Electrodiagnosis of Martin-Gruber connection and its clinical importance in peripheral nerve surgery. J Hand Surg Am 1992;17:54–9. 1311344 10.1016/0363-5023(92)90113-4

[5] Wichlas F, Haas NP, Disch A, Machó D, Tsitsilonis S. Complication rates and reduction potential of palmar versus dorsal locking plate osteosynthesis for the treatment of distal radius fractures. J Orthop Traumatol 2014;15:259–64. 25027735 10.1007/s10195-014-0306-y PMC4244564

[6] Lutz K, Yeoh KM, MacDermid JC, Symonette C, Grewal R. Complications associated with operative versus nonsurgical treatment of distal radius fractures in patients aged 65 years and older. J Hand Surg Am 2014;39:1280–6. 24881899 10.1016/j.jhsa.2014.04.018

[7] Yu YR, Makhni MC, Tabrizi S, Rozental TD, Mundanthanam G, Day CS. Complications of low-profile dorsal versus volar locking plates in the distal radius: a comparative study. J Hand Surg Am 2011;36:1135–41. 21712136 10.1016/j.jhsa.2011.04.004

[8] Nourbakhsh A, Tan V. Median nerve fibrosis at the distal forearm after volar plate fixation of distal radius fracture. J Hand Surg Eur 2010;35:768–9. 10.1177/1753193410379547 20974882

[9] Arora R, Lutz M, Deml C, Krappinger D, Haug L, Gabl M. A prospective randomized trial comparing nonoperative treatment with volar locking plate fixation for displaced and unstable distal radial fractures in patients sixty-five years of age and older. J Bone Joint Surg Am 2011;93:2146–53. 22159849 10.2106/JBJS.J.01597

[10] Jakubietz MG, Gruenert JG, Jakubietz RG. Palmar and dorsal fixed-angle plates in AO C-type fractures of the distal radius: is there an advantage of palmar plates in the long term? J Orthop Surg Res 2012;7:8. 22340861 10.1186/1749-799X-7-8 PMC3312832

[11] Wilcke MK, Abbaszadegan H, Adolphson PY. Wrist function recovers more rapidly after volar locked plating than after external fixation but the outcomes are similar after 1 year. Acta Orthop 2011;82:76–81. 21281262 10.3109/17453674.2011.552781 PMC3230001

[12] Lauder A, Agnew S, Bakri K, Allan CH, Hanel DP, Huang JI. Functional outcomes following bridge plate fixation for distal radius fractures. J Hand Surg Am 2015;40:1554–62. 26143028 10.1016/j.jhsa.2015.05.008

[13] Ginn TA, Ruch DS, Yang CC, Hanel DP. Use of a distraction plate for distal radius fractures with metaphyseal and diaphyseal comminution. Surgical technique J Bone Surg Am 2006;88:29–36. 16510798 10.2106/JBJS.E.01094

